# Tumor-infiltrating lymphocytes predict prognosis of breast cancer patients treated with anti-Her-2 therapy

**DOI:** 10.18632/oncotarget.14124

**Published:** 2016-12-23

**Authors:** Tan-Huan Chen, Ying-Chun Zhang, Yu-Ting Tan, Xin An, Cong Xue, Ying-Fei Deng, Wei Yang, Xia Yuan, Yan-Xia Shi

**Affiliations:** ^1^ Department of Medical Oncology, Affiliated Hui Zhou Municipal Central Hospital & Training Base for Masters of Sun Yat-Sen Memorial Hospital, Sun Yat-Sen University, Huicheng District, Huizhou, Guangdong 516000, P. R. China; ^2^ Department of Medical Oncology, Sun Yat-Sen University Cancer Center, Guangzhou, Guangdong 510000, P.R. China; ^3^ Department of Pathology, Sun Yat-Sen University Cancer Center, Guangzhou, Guangdong 510000, P.R. China; ^4^ Department of Radiotherapy, Sun Yat-Sen University Cancer Center, Guangzhou, Guangdong 510000, P.R. China

**Keywords:** TILs, anti-Her-2 therapy, breast cancer, prognosis

## Abstract

**Purpose:**

Infiltration of tumor associated lymphocytes and count of its different phenotypes are potentially new independent predictor of prognosis in breast cancer. However, research related to it is less reported in breast cancer patients treated with anti-Her-2 therapy. Thus, we evaluated the relationship between survival and tumor infiltrating lymphocytes including its different phenotypes in tumors of such patients.

**Methods:**

Between 1999 and 2010, 98 patients diagnosed with primary breast cancer and treated with anti-Her-2 therapy at Sun-Yat-Sen University Cancer Center were included in the study. Biopsy specimens were collected post-operation but before chemotherapy. Tumor infiltrating lymphocytes as well as its FOXP3+, CD68+, IL-17+ phenotypes in both intratumoral and stromal sites and expression of FOXP3 in cancer cells were assessed.

**Results:**

Median follow-up time of 98 patients was 83.3 months (range 7.4-201 months). It suggested that patients with high stromal infiltration of TILs, lower count of FOXP3+ Tregs and CD68+ Mφ in stromal site, and high expression of FOXP3 in cancer cells had longer survival of OS. In multivariate Cox regression analysis, high count of intratumoral CD68+ Mφ [HR: 2.70 (1.00–7.31); p=0.050] and high expression of FOXP3 in cancer cells [HR: 0.29 (0.09–0.91); p=0.034] were independent prognostic factors for overall survival.

**Conclusions:**

Tumor infiltrating lymphocytes as well as its FOXP3+, CD68+ phenotypes in stromal site, and expression of FOXP3 in cancer cells were significantly associated with OS, suggesting that they can be used as important pathological factor predicting prognosis of breast cancer patients treated with anti-Her-2 therapy.

## INTRODUCTION

Breast cancer was the mostly diagnosed tumor for women worldwide now, and incidence of it kept on increasing year by year. For tumor related death of women, breast cancer was the second causing disease too [1]. In all of the breast cancer patients, 25-30% were with Her-2 (human epidermal growth factor receptor-2) proto-oncogene amplification or excessive expression of Her-2 protein. Her-2 status was also one of the most important prognostic factor in breast cancer and overexpression with Her-2 was associated with disease progress and prognosis of patients [2]. Anti-Her-2 therapy which was mainly represented by trastuzumab can not only significantly inhibit tumor growth but also synergize with traditional cytotoxic chemotherapy to reduce recurrence risk of operable breast cancer patients by 46% and prolonged the OS (overall survival) of advanced breast cancer patients by 5-15 months [3].

Being the basis of Her-2 positive breast cancer treatment, trastuzumab was a humanized monoclonal antibody targeting at extracellular domain of Her-2, and previous studies demonstrated blocking Her-2 mediated signaling pathway was the main mechanism of its efficacy. However, the recent research found that trastuzumab can induce antibody dependent cellular cytotoxicity (ADCC) and play an immunomodulatory role in the course of the anti-Her2 therapy which were crucial to its efficacy [4, 5]. Meanwhile, on the tumor response to chemotherapy, immune cells, especially tumor infiltrating lymphocytes (TILs) in tumor microenvironment and its potential role attracted more and more attention [6]. More and more evidence showed that the interaction between these immune cells and tumor was important for the course and progress of tumor [7] and related to efficacy of trastuzumab [8–12]. Several studies also indicated that TILs can predict better response of higher pathologic complete response (pCR) rate to chemotherapy and anti-Her-2 therapy in breast cancer [6, 13–17].

Tumor-infiltrating lymphocytes(TILs) were white blood cells that left the bloodstream and migrated into a tumor. They were mononuclear immune cells, a mix of different types of cells (i.e., T cells, B cells, NK cells, macrophages) in variable proportions [18]. As the natural anti-tumor immune barrier of host, monocyte-macrophage(Mφ) were important component of TILs. In normal tissues, Mφ showed spontaneous anti-tumor effect [19, 20]. As the most abundant antigen-presenting cells in solid tumor, Mφ expressed FCγR (Fc-gamma receptor) on its surface and by combining with FCγR trastuzumab can induce ADCC to surpress tumor [21]. However, there was another unique subtype of Mφ described as M2 which can suppress antitumor immunity and promote tumor progression [22, 23]. Thus, Mφ were a highly heterogeneous group of cells that maybe play different functions in different tumor microenvironment and therefore efficacy of trastuzumab may varied with different type of Mφ.

Trastuzumab can induce the production of endogenous anti-Her-2 antibody and antigen-specific CD4+ T cells by activating antigen-specific humoral immunity in vivo. Clare Taylor et al reported after 8 weeks of treatment combining trastuzumab with chemotherapy, endogenous anti-Her-2 antibody and antigen-specific CD4+ T cells can be detected in the peripheral blood circulation, and this immune response can be sustained through 15 weeks and brought benefit to patients with prolonged PFS (progression free survival) [24]. Besides, CD8+ T cell mediated cellular immunity also played an important role in anti-tumor immunology process through its cytotoxic effect. And Park S et al reported crucial role of T cell in trastuzumab treatment [8]. It showed that efficacy for inhibiting tumor growth of anti-Her-2 antibody weakened greatly in the mice lack of T cells and elimination of CD8 + T cells in wild type mice significant promoted tumor recurrence. On the contrary, in tumor tissue of mice and patients treated with anti-Her-2 antibodies, T cells, especially CD8+ T cells can be increasingly detected. And these existing and more effector and memory T cells maybe make the mice to tolerate higher doses of tumor cells inoculation thereafter. Moreover, in Rag−/− immunodeficiency mice with specifical elimination of T and B cells, curative effect of trastuzumab was very weak, indicating the mechanism of effect for trastuzumab largely depended on humoral immunity and cellular immunity of host.

*In vivo*, both CD4+ T and CD8+ T cells were regulated by CD4+, CD25+ regulatory T cells (Tregs) whose specific marker was FOXP3 (forkhead box P3) [25]. Mφ and tumor in situ can secrete IL-10 to recruit Tregs to tumor site [26] indicating that Mφ may affect the number of FOXP3+ Tregs by cytokines. Tregs would increase in tumor site or be induced into CD4+, CD25+ T cells by tumor-associated DCs (dendritic cells) [27–29] and FOXP3 gene played a critical role in Tregs’ differentiation, development and maintenance of function during this process [30].

Th1 (T helper 1 cells) produced IFN-γ(interferon gamma) to promote anti-microbial and anti-tumor response, and Tregs suppressed the immune response of T cells in both physiological and pathological state [31, 32]. Another new CD4+ T helper cells can produce IL-17 was defined as Th17 (T helper 17 cells), and most of the inflammatory damage which before was thought to be caused by Th1 response now was found to be caused by IL-17 and IL-23, which were important cytokines in vivo that can support Th17 reaction [33, 34]. However, the role of Th17 in human tumor progression was still not clear.

Studies also reported that trastuzumab can reduce Tregs in peripheral circulation and therefore broke the balance between Tregs and Th17 [35] so that eliminated the deactivated status of immune system to promote the host's anti-tumor immunity. Therefore, anti-tumor effect of trastuzumab may be a complex but orderly process which firstly released detrimental factors by blocking Her-2 mediated signal transduction pathways and then activated hosts’ innate and adaptive immune response including humoral and cellular immunity to control and eliminate tumors.

Many researchers explored prognostic value of Her-2 gene amplification and protein overexpression to anti-Her-2 therapy. However, Perez et al found 174 patients who were Her-2 negative detected by IHC (immunohistochemistry) and FISH (fluorescence in situ hybridization) also benefited from anti-Her-2 therapy (DFS HR 0.34; 95% CI, 0.14-0.80; P = 0.014) [36]. Besides, in pathways linked to Her-2, molecular and genetic factors such as NRGs (neuregulins) [37], IGF-1R(insulin-like growth factor 1 receptor) [38], PI3K (phosphoinositide 3-kinase), PTEN (phosphatase and tensin homolog), mTOR (mechanistic target of rapamycin) and NF-κB (nuclear factor kappa-light-chain-enhancer of activated B cells) [39] were all potentially or provenly associated with the signal transduction pathway of Her-2 and consequently therapeutic effect of anti-Her-2 therapy. Nevertheless, predictive value of these factors to prognosis reported by literatures was inconsistent, and therefore was far away from clinical application.

In view of the value of immune factors for anti-Her-2 therapy, the factors that affect the immune response such as TILs, are likely to influence the curative effect of anti-Her-2 therapy [6, 40]. Studies indicated that including affinity and polymorphism of FCγR receptor on immune effector cells [21], host immune status and local immune status around tumor such as ratio of immunosuppressive regulatory cells around tumor or in peripheral circulation which is represented by the ratio of Tregs/Th17, pDC/mDC (plasmacytoid dendritic cell/ myeloid dendritic cell), etc [41], and chemotherapeutic agents used in combination with trastuzumab [8], all these factors can affect the immune response. As a result, whether these baseline immune index and strength of host's adaptive immune response can work as a predictor of efficacy of anti-Her-2 therapy attracted more interest and attention, taking into account its crucial significance for patients without measurable lesions and received adjuvant therapy.

In conclusion, in this study, by detecting quantity and distribution of FOXP3+ Tregs, CD68+ Mφ and IL-17+ Th17 in the tumor microenvironment as well as potential relationship between them, we explored the value of these factor to predict efficacy of anti-Her-2 therapy, attempting to guide treatment options for anti-Her-2 therapy.

## RESULTS

### Patient characteristics

A total of 98 patients treated with anti-Her-2 therapy were included in the present study. The median age at diagnosis was 47 years (range, 26–76 years) (Table 1). All of the patients were female and median follow-up time is 83.3 months (range 7.4-201 months). Although 14 patients were Her-2/neu negative by IHC (FISH test result unavailable) or unspecified (both IHC and FISH results unavailable), we still included them at last according to their records of treatment with anti-Her-2 therapy (trastuzumab). Clinical characteristics of 98 patients were showed in Table 1.

**Table 1:** Characteristics of 98 breast cancer patients treated with anti-Her-2 therapy

Characteristic	n (%)
Age at diagnosis, years	
Median age	47 (Range 26~76)
>50	41(42%)
≤50	57(58%)
Menopausal status	
Pre	56(57%)
Post	42(43%)
Tumor size	
>2cm	51(52%)
≤2cm	32(33%)
Unavailable	15(15%)
Nodal status	
N_0_	25(26%)
N_1_	29(30%)
N_2_	22(22%)
N_3_	12(12%)
Unavailable	10(10%)
Estrogen receptor status	
Positive	35(36%)
Negative	63(64%)
Progestrone receptor status	
Positive	53(54%)
Negative	45(16%)
HER2/neu status(IHC)	
Positive	84(86%)
Negative^A^	12(12%)
Unavailable^B^	2(2%)
TNM stage at diagnosis	
I	9(9%)
II	35(36%)
III	34(35%)
IV	17(17%)
Unavailable	3(3%)
Intratumoral TILs	
<10%	82(84%)
≥10%	16(16%)
Stromal TILs	
<10%	41(42%)
≥10%	57(58%)
Chemotherapy regimen^C^	
A/T	46(47%)
A+T	45(46%)
others	7(7%)

Abbreviation: TILs, tumor-infiltrating lymphocytes; A/T, anthracycline or taxol based regimen; A+T, anthracycline and taxol based regimen; A: FISH test result was unavailable; B: both IHC and FISH results were unavailable; C: all regimens included anti-Her-2 therapy (trastuzumab);

### Histopathological and immunohistochemical evaluation

**Figure 1:**
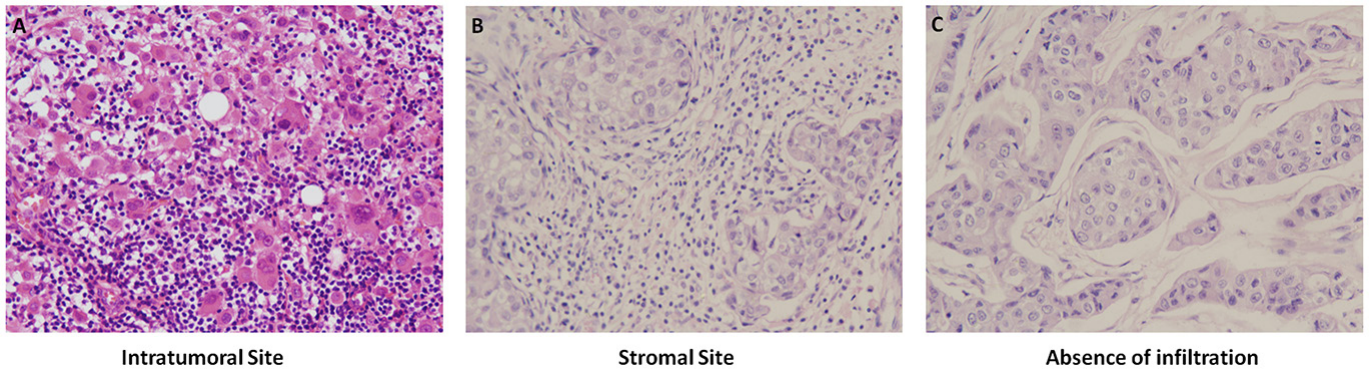
Richness and absence of TILs in intratumoral and stromal sites by HE staining, 1×200

In 98 HE stained breast cancer tissue samples: Median TILs percent of intratumoral site was 3% (range 0.5%-90%), with 16 patients ≥10% and 82 patients <10%. Median TILs percent in stromal site was 10% (range 2.5%-90%), with 57 patients ≥10% and 42 patients <10%. Richness and absence of TILs in intratumoral and stromal sites by HE staining were showed in Figure 1.

**Figure 2:**
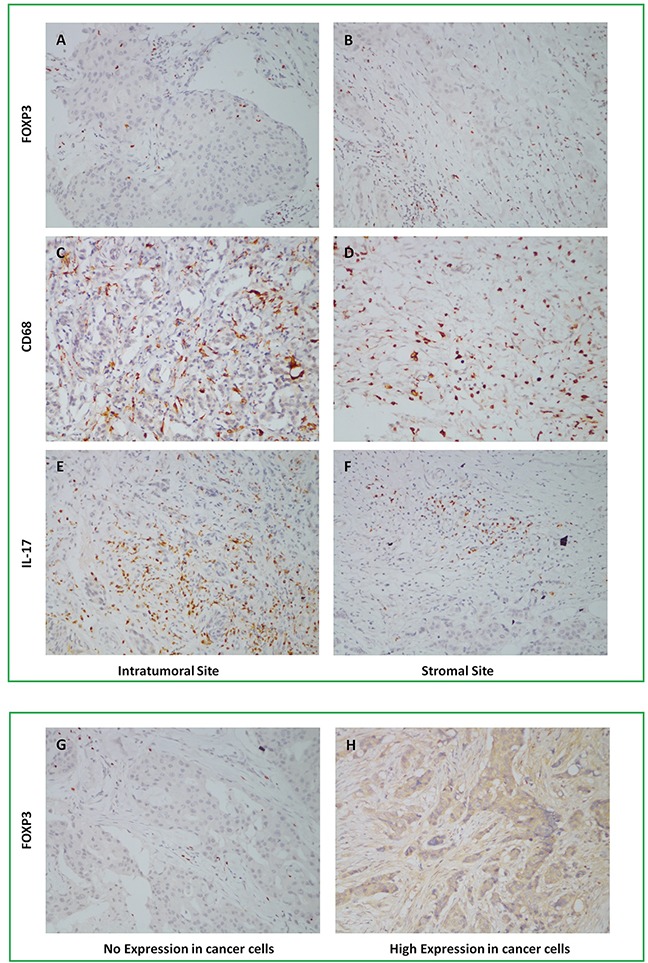
Infiltration of FOXP3+ Tregs, CD68+ Mφ, IL-17+ Th17 in intratumoral and stromal sites, and Foxp3 expression in cancer cells by IHC staining, 1×200

For IHC staining, the positive expression of FOXP3+ Tregs was pale brown in cell nucleus with the median value of positive cells count in intratumoral site 6.8/HPF (range 3-18.3/HPF) and stromal site 17.2/HPF (range 6-94.7/HPF). Infiltration of FOXP3+ Tregs in intratumoral and stromal site were showed in Figure 2. The positive expression of CD68+ Mφ was brown in cytoplasm with the median value of positive cells count in intertumoral site 66/HPF (range 12-98.7/HPF) and stromal site 81.3/HPF (range 26.3-131.3/HPF). Infiltration of CD68+ Mφ in intratumoral and stromal site were showed in Figure 2. The positive expression of IL-7+ Th17 was pale brown in cytoplasm with the median value of positive cells count in intratumoral site 1.3/HPF (range 0.3-9.3/HPF) and stromal site 4.3/HPF (range 0.7-13.7/HPF). Infiltration of IL-7+ Th17 in intratumoral and stromal site were showed in Figure 2. The results of FOXP3+ Tregs, CD68+ Mφ and IL-17+ Th17 counts were presented in Supplementary Table S1.

In addition to TILs, expression of FOXP3 was also found in tumor cells and two independent reviewers evaluated it by percentage of positive tumor cells of total tumor cells. Average of two reviewers’ value was the final result and expression percentage>median was defined as high expression of FOXP3 in tumor cells. Median of FOXP3 expression in tumor cells was 3% (range 0-85%) with 39 patients high expression and 59 patients low expression. Expression of FOXP3 in cancer cells was showed in Figure 2. Correlation between infiltration of TILs, three different phenotypes of TILs and clinicopathological features of 98 patients was showed in Table 2.

**Table 2:** Correlation between infiltration of TILs, three different phenotypes of TILs and clinicopathological features of patients

Variables	TIL						FOXP3						CD68						IL-17					
	IS			SS			IS			SS			IS			SS			IS			SS		
	H	L	P	H	L	P	H	L	P	H	L	P	H	L	P	H	L	P	H	L	P	H	L	P
Age(years)																								
>50	8	33	**0.33**	24	17	**0.56**	19	22	**0.34**	19	22	**0.41**	18	23	**0.21**	20	21	**0.57**	24	17	**0.33**	23	18	**0.26**
≤50	8	49		33	24		30	27		29	28		31	26		28	29		37	20		27	30	
Menopausal status																								
Pre	9	47	**0.57**	32	24	**0.49**	29	27	**0.42**	28	28	**0.49**	29	27	**0.42**	27	29	**0.51**	35	21	**0.56**	26	30	**0.20**
Post	7	35		25	17		20	22		20	22		20	22		21	21		26	16		24	18	
Tumor Size																								
>2cm	9	42	**0.56**	31	20	**0.54**	26	25	**0.55**	22	29	**0.25**	25	26	**0.45**	23	28	**0.42**	35	16	**0.12**	29	22	**0.11**
≤2cm	6	26		19	13		16	16		17	15		17	15		16	16		17	15		13	19	
UA	1	14		7	8		7	8		9	6		7	8		9	6		9	6		8	7	
Node status																								
N_3_+N_2_	6	28	**0.58**	22	12	**0.39**	14	20	**0.14**	18	16	**0.29**	15	19	**0.38**	18	16	**0.23**	24	10	**0.09**	20	14	**0.10**
N_1_+N_0_	10	44		32	22		30	24		24	30		27	27		23	31		29	25		23	31	
UA	0	10		3	7		5	5		6	4		7	3		7	3		8	2		7	3	
ER status																								
+	3	32	**0.10**	21	14	**0.48**	17	18	**0.50**	16	19	**0.39**	20	15	**0.20**	15	20	**0.24**	18	17	**0.08**	17	18	**0.44**
−	13	50		36	27		32	31		32	31		29	34		33	30		43	20		33	30	
PR status																								
+	8	45	**0.47**	34	19	**0.14**	21	32	**0.02**	22	31	**0.08**	28	25	**0.34**	21	32	**0.04**	31	22	**0.27**	27	26	**0.57**
−	8	37		23	22		28	17		26	19		21	24		27	18		30	15		23	22	
Her-2 status																								
+	15	69	**0.11**	50	34	**0.20**	41	43	**0.20**	41	43	**0.59**	41	43	**0.38**	39	45	**0.16**	55	29	**0.10**	40	44	**0.18**
−	0	12		5	7		8	4		6	6		7	5		8	4		5	7		8	4	
UA	1	1		2	0		0	2		1	1		1	1		1	1		1	1		2	0	
TNM-stages																								
IV	0	17	**0.03**	5	12	**0.01**	9	8	**0.48**	10	7	**0.31**	11	6	**0.15**	11	6	**0.13**	14	3	**0.05**	10	7	**0.35**
III+II+I	16	62		50	28		38	40		38	40		37	41		36	42		45	33		39	39	
UA	0	3		2	1		2	1		0	3		1	2		1	2		2	1		1	2	

Abbreviation: TILs, tumor-infiltrating lymphocytes; IS, intratumoral site; SS, stromal site; H, high level; L, low level; P, p-value; UA, unavailable;

### Survival analysis

**Figure 3:**
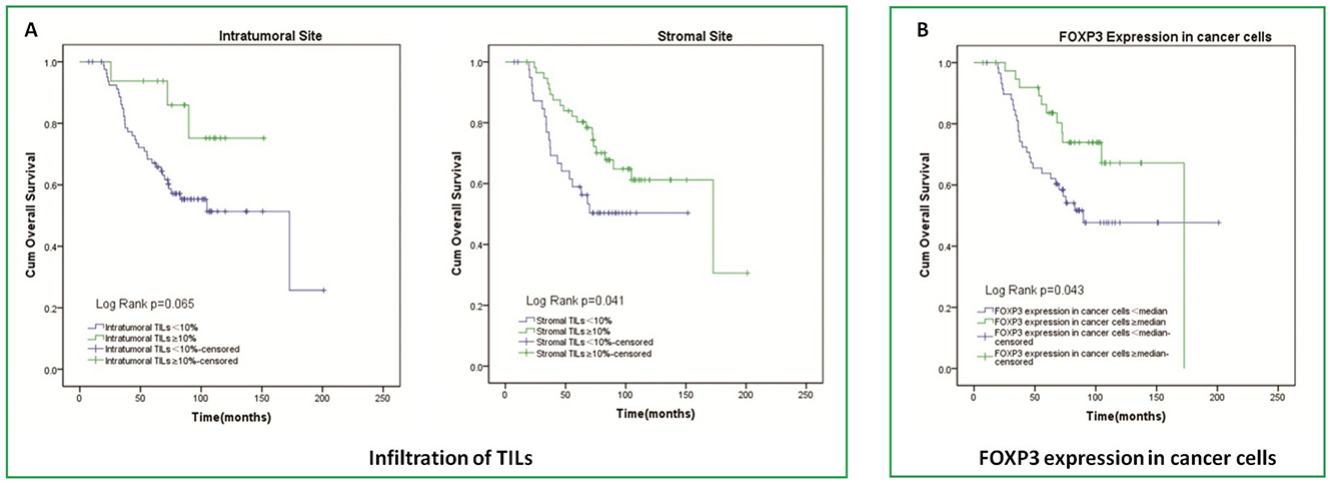
Kaplan-Meier curves of overall survival according to TILs infiltration in intratumoral and stromal sites and FOXP3 expression in cancer cells

In survival analysis, with 16 patients ≥10% linked to survival of 130.9 months (range 110.0-151.8 months) and 82 patients <10% linked to survival of 120.7 months (range 101.4-140.1 months), patients with high infiltration of intratumoral TILs just tended to had longer OS than patients with low, because of the p-value of 0.065; On the other side, with 57 patients ≥10% linked to survival of 138.0 months (range 116.5-159.4 months) and 41 patients <10% linked to survival of 96.2 months (range 78.1-114.2 months), patients with high infiltration of stromal TILs also had longer OS than patients with low, and data of two groups are statistically significant with p=0.041 (Figure 3).

**Figure 4:**
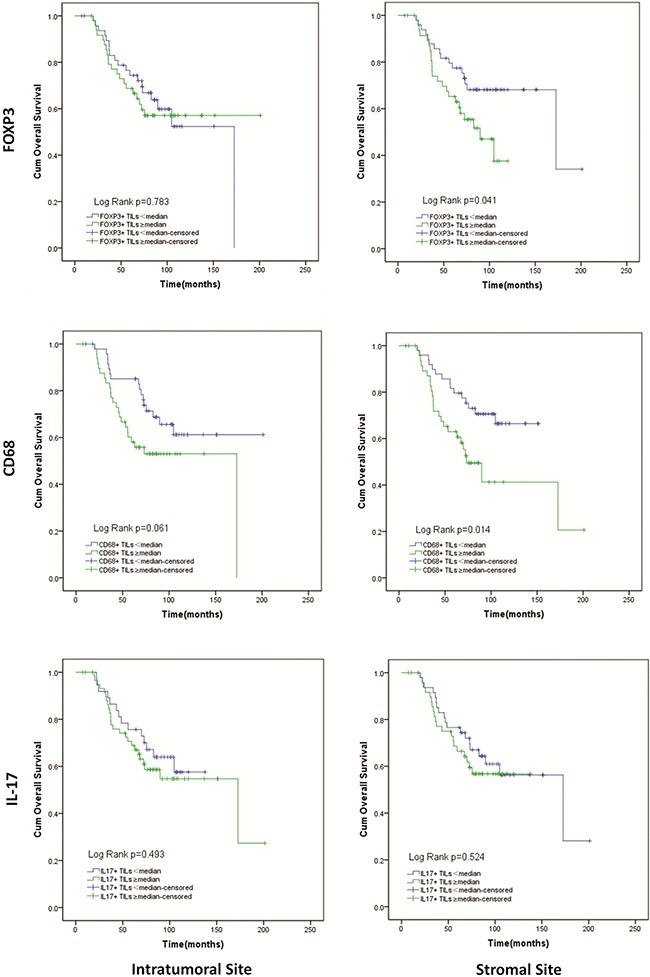
Kaplan-Meier curves of overall survival according to infiltration of FOXP3+ Tregs, CD68+ Mφ, IL-17+ Th17 in intratumoral and stromal sites

Patients with a lower number of FOXP3+ Tregs in stromal site had significantly longer OS than patients with a higher number of stromal FOXP3+ Tregs (142.6 (119.5–165.6) vs. 81.3(70.1–92.6) months; p=0.041). However, for intratumoral site, there was no difference between patients with higher and lower FOXP3+ Tregs in OS (Figure 4). A higher number of CD68+ Mφ in intratumoral site tended to result in shortened OS (111.0(91.5–130.5) vs. 147.3(126.0–168.6) months; p=0.061). And similar to intratumoral site, patients had higher stromal CD68+ Mφ count had shorter OS (106.6(81.3–132.0) vs. 120.0(106.4–133.5) months; p=0.014) (Figure 4). For IL-17+ Th17, there was just a trend that in stromal sites higher IL-17+ Th17 count tended to result in shorter OS (97.6(83.9–111.2) vs. 130.3(107.1–153.5) months; p=0.524). However, in intratumoral site, result of IL-17+ Th17 seemed to conflictive (Figure 4). We also evaluated the effect of FOXP3 expression in tumor cells on overall survival and observed that patients with high level of FOXP3 expression in tumor cells survived a longer time than those with low level of FOXP3 expression. (137.2(117.6–156.8.) vs. 120.7(99.2–142.2) months; p=0.043) (Figure 3).

### Cox-regression analysis

The factors found to have an impact on survival (or considered to be effective) including ages at diagnosis, menopausal status, tumor size, nodal status, ER status, PR status, TNM stages, FOXP3+ expression in cancer cells and TILs, and FOXP3+ Tregs, CD68+ Mφ, IL-17+ Th17 in both intratumoral and stromal sites were re-analyzed using Cox regression analyses. In univariate model, nodal status of N_3_ and N_2_ [HR: 3.28 (1.54–6.95); p=0.002], TNM stages of IV [HR: 4.64 (2.34–9.20); p=0.000], stromal TILs≥10% [HR: 0.52 (0.27–0.99); p=0.045], high count of stromal FXOP3+ Tregs [HR: 1.95 (1.01–3.75); p=0.045], high count of stromal CD68+ Mφ [HR: 2.24 (1.16–4.34); p=0.017] and high expression of FOXP3 in cancer cells [HR: 0.49 (0.25–0.99); p=0.048] had an independent effect on the risk of death (Table 3).

**Table 3:** Univariate analysis of factors associated with overall survival

Variable	HR	95% CI	P-value
Age at diagnosis (y>50/y≤50)	1.49	0.79-2.82	**0.218**
Menopausal status (Pre/Post)	0.66	0.35-1.23	**0.192**
Tumor size (>2cm/≤2cm)	0.55	0.27-1.14	**0.109**
Nodal status (N_3_+N_2_/N_1_+N_0_)	3.28	1.54-6.95	**0.002**
ER status (+/−)	0.67	0.34-1.34	**0.260**
PR status (+/−)	0.64	0.34-1.20	**0.163**
TNM stages (IV/III+II+I)	4.64	2.34-9.20	**0.000**
Intratumoral TILs (≥10%/<10%)	0.35	0.11-1.13	**0.078**
Stromal TILs (≥10%/<10%)	0.52	0.27-0.99	**0.045**
Intratumoral FXOP3+ Tregs (high/low)	1.09	0.58-2.05	**0.783**
Stromal FXOP3+ Tregs (high/low)	1.95	1.01-3.75	**0.045**
Intratumoral CD68+ Mφ(high/low)	1.83	0.96-3.49	**0.065**
Stromal CD68+ Mφ(high/low)	2.24	1.16-4.34	**0.017**
Intratumoral IL17+ Th17 (high/low)	1.26	0.65-2.44	**0.494**
Stromal IL17+ Th17 (high/low)	1.23	0.65-2.33	**0.525**
FOXP3+ cancer cells (high/low)	0.49	0.25-0.99	**0.048**

Abbreviation: HR, hazard ratio; CI, confidence interval; ER, estrogen receptor; PR, progestrone receptor; TILs, tumor-infiltrating lymphocytes; Tregs, regulatory T cells; Mφ, macrophage; Th17, T helper cell 17;

Multivariate analysis was performed using factors which had or tended to have independent effect in univariate analysis and other basically clinical factors of patients. Multivariate analysis showed that age >50 years [HR: 0.03 (0.00–0.56); p=0.018], pre-menopausal [HR: 0.02 (0.00–0.29); p=0.005], tumor size >2cm [HR: 0.15 (0.05–0.47); p=0.001], nodal status of N3+N2 [HR: 6.39 (1.90–21.5); p=0.003], TNM stages of IV [HR: 5.36 (1.33–21.5); p=0.018], high count of intratumoral CD68+ Mφ [HR: 2.70 (1.00–7.31); p=0.050] and high expression of FOXP3 in cancer cells [HR: 0.29 (0.09–0.91); p=0.034] were independent prognostic factors of OS (Table 4).

**Table 4:** Multivariate analysis of factors associated with overall survival

Variable	HR	95% CI	P-value
Age at diagnosis (y>50/y≤50)	0.03	0.00-0.56	**0.018**
Menopausal status (Pre/Post)	0.02	0.00-0.29	**0.005**
Tumor size (>2cm/≤2cm)	0.15	0.05-0.47	**0.001**
Nodal status (N_3_+N_2_/N_1_+N_0_)	6.39	1.90-21.5	**0.003**
ER status (+/−)	0.65	0.24-1.75	**0.390**
PR status (+/−)	1.50	0.53-4.21	**0.442**
TNM stages (IV/III+II+I)	5.36	1.33-21.5	**0.018**
Intratumoral TILs (≥10%/<10%)	0.32	0.06-1.56	**0.157**
Stromal TILs (≥10%/<10%)	1.12	0.41-3.08	**0.831**
Stromal FXOP3+ Tregs (high/low)	1.34	0.53-3.38	**0.534**
Intratumoral CD68+ Mφ(high/low)	2.70	1.00-7.31	**0.050**
Stromal CD68+ Mφ(high/low)	1.85	0.72-4.73	**0.200**
FOXP3+ cancer cells (high/low)	0.29	0.09-0.91	**0.034**

Abbreviation: HR, hazard ratio; CI, confidence interval; ER, estrogen receptor; PR, progestrone receptor; TILs, tumor-infiltrating lymphocytes; Tregs, regulatory T cells; Mφ, macrophage;

## DISCUSSION

In this retrospective analysis including 98 breast cancer patients treated with anti-Her-2 therapy, we studied potential relationship between TILs infiltration, count of FOXP3+ Tregs, CD68+ Mφ and IL-17+ Th17 in both intratumoral and stromal site with patients’ survival. It suggested that patients with high stromal infiltration of TILs, lower count of FOXP3+ Tregs and CD68+ Mφ in stromal site, and high expression of FOXP3 in cancer cells had longer survival of OS. In multivariate Cox regression analysis, high count of intratumoral CD68+ Mφ and high expression of FOXP3 in cancer cells were independent prognostic factors for overall survival.

Although the precise mechanism remained unclear, the usual explanation was that different tumor microenvironment may induce Mφ having specific functions to facilitate different tumor cell activities [42]. In addition to immune suppression, Mφ can release cytokines to regulate tumor growth, angiogenesis, invasion and metastasis. Relationship between Mφ and tumor cells was important for vascular invasion of tumor cells within the primary tumor [26, 42]. Such a mechanism may be used to explain the observation that higher macrophage density was associated with poor prognosis. Studies focused in HCC (hepatocellular carcinoma) found that most of the Mφ was unique phenotype with expression of low HLA-DR (human leukocyte antigen-antigen D related) and high IL-10 in the tumor nests but another different phenotype with expression of moderate HLA-DR and negative IL-10 in the peritumoral stromal region [26]. It indicated local microenvironment around tumor nests may promote functional translation of Mφ to immunosuppression and tumor-promotion.

Immunosuppression mediated by Tregs was thought to be key reason for tumor immune escape and main obstacle to success of tumor immunotherapy [27]. Soluble cytokines from the tumor microenviroment, especially cytokines secreted by tumor cells and APCs (antigen presenting cells), can induce aggregation, proliferation and migration of Tregs [27]. Previous studies reported that as component of peripheral blood circulation and TILs, increased Tregs can weaken cell-mediated immunity therefore promoted disease progression in ovarian cancer or esophageal and gastric cancer [43–45]. Studies showed that elimination of Tregs in melanoma mouse model can increase the effectiveness of immunotherapy and disappearance rate of tumor lesions [46, 47]. Moreover, elimination of CD4+&CD25+ Tregs in advanced cancer patients promoted efficacy of T cells and NK cells [48, 49]. These evidences suggested that Tregs can destruct cell-mediated immune response to tumor. Zheng et al found increasement of intratumoral FOXP3+ Tregs was linked to shorter OS and DFS and was independent prognostic factor in HCC patients [50]. Bohling and Allison found density of intratumoral FOXP3+ Tregs was significantly associated with high histological grade, larger size and ER negative status in breast cancer [51]. This also supported our study that higher infiltration of FOXP3+ Tregs in stromal site was associated with poor prognosis in breast cancer patients, although no significant difference was found when it comes to intratumoral site.

Studies also reported that many tumor cell lines including lung cancer, colon cancer, breast cancer, melanoma, acute T-cell leukemia cell lines can express FOXP3 protein [52]. Coincidentally, in this study, in addition to FOXP3+ Tregs we also found that there were tissue samples expressing FOXP3 protein in tumor cells. In 103 breast cancer tissue samples stained with FOXP3 antibody by IHC, Ladoire et al found 57% of tumors expressing different levels of FOXP3 protein and as independent prognostic factor, high level of FOXP3 protein expression was associated with prolonged DFS and OS [53]. Zuo et al also compared expression of FOXP3 and Her-2 protein by IHC in breast cancer tissue and found downregulation of FOXP3 protein was significantly with overexpression of Her-2 protein [54]. They also confirmed that FOXP3 was X-linked and was important suppressor gene of both breast cancer and Her-2/ErbB2 proto-oncogene [54]. Other finds were including but not only that 80% of the normal epithelial cells in breast tissue expressed FOXP3 and only 20% of breast cancer tissue expressed it by IHC [54]. Thus, it can be explained that in our study high expression of FOXP3 in cancer cells was associated with prolonged OS.

Studies reported TNF-β(tumor necrosis factorβ) played a key role in determining the differentiation of CD4+ lymphocytes to be Tregs or Th17 [55, 56]. Th17 were defined as one of the subtype of CD4+ lymphocytes characterized in that Th17 can produce IL-6, IL-17 and TNF-α, etc. Differentiation of Th17 was induced by IL-23 which was also inducer of autoimmune encephalitis [57, 58]. Balance between Tregs and Th17 may affect the anti-tumor immune response and more Tregs tended to result in tumor's immune escape. Recently studies suggested that IL-17 and its producing cells Th17 were disadvantageous factors in breast cancer by changing the behavior of tumor cells, eliciting tumorigenic neutrophils recruitment [59], promoting chemoresistance, proliferation of tumor cells [60], tumor angiogenesis [61] and metastasis [62]. Moreover, by flow cytometry, Horlock et al observed that both Th17 and FOXP3+ Tregs increased in peripheral blood of breast cancer patients and with the treatment of trastuzumab, Th17 increased but FOXP3+ Tregs decreased which led to changes of Th17/Tregs ratio [35]. Such dynamic balance between Th17 and Tregs can reflect even predict the efficacy of trastuzumab [35]. However, we did not find obvious relationship between Th17 and prognosis or relationship between Th17 and anti-Her-2 therapy probably due to relatively less expression of IL-17 in this study.

Furthermore, study showed that the delayed use of trastuzumab has no negative effect on the OS of Her-2 positive advanced breast cancer patients and there is a trend of improved OS over the patients with repeated use of trastuzumab [63]. This evidence further supported that anti-Her2 therapy is closely associated with immune system, because it can't be fully explained that trastuzumab still had effect after tumor progression just by blocking Her-2 signaling pathway unless it promote the anti-tumor immune response by long-term or repeated use of trastuzumab. It suggested on the other hand that infiltration of TILs and its different phenotype in tumor microenvironment affect the efficacy of anti-Her2 therapy.

In addition, at the genetic level related genes associated with TILs, FOXP3, CD68 and IL-17 were also reported. By comparing the gene expression pattern of TILs with reported data and UniGene, Li, B. et al demonstrated the parallel gene expression of TILs had an important role in T-cell activity, infiltrating of TILs within tumor tissue and photokilling effect against tumor cells of TILs [64]. There was another first-in-man trial showing that administration of TILs with transduction of an inducible IL12 gene can mediate tumor responses [65]. Evidences indicated CD8+ TILs can be activated by IL-2 secreted by TILs and IL-2 gene expression may be an available prognostic factor in HCC [66]. It seemed that IL-17 gene maybe had a similar function and mechanism. However, the difference was IL-17 gene polymorphism. E. LBassuoni MA et al showed the GG, GG+GA genotypes of IL-17A gene promoted the development of HCC through increased IL17 and IgE [67]. Higher immune gene expression of IL-17 was also been proven to be a determinant in mismatch repair proficient colorectal cancer, and so was FOXP3 [68]. In studies of nearly two years, genome-wide analysis of Foxp3 expression in tongue squamous cell carcinoma cells revealed that Foxp3 gene had more significant biological effects in tumor cells compared with that in FOXP3+ Tregs and consequently demonstrated diverse genes that FOXP3 gene directly or indirectly targeted in tumor cells [69]. As a suppressor gene in breast cancer cells, there was also research showing that interaction of Runx1 and FOXP3 genes can affected gene expression profile of mammary epithelial cell gene and finally Runx1 cause breast cancer progression on FOXP3 availability [70]. For CD68, Tymoszuk, P. et al showed that the higher expression of related gene STAT1 was related to increased expression of tumor-associated macrophages gene such CD68 and led to poor prognosis [71].

In conclusion, the retrospective study including 98 breast cancer patients treated with anti-Her-2 therapy revealed that tumor TILs as well as its FOXP3+, CD68+ phenotypes in stromal site, and expression of FOXP3 in cancer cells were significantly associated with OS. Patients with high stromal infiltration of TILs, lower count of FOXP3+ Tregs and CD68+ Mφ in stromal site, and high expression of FOXP3 in cancer cells had longer OS. And high count of intratumoral CD68+ Mφ and high expression of FOXP3 in cancer cells were independent prognostic factors for overall survival. It suggested that TILs and its specific subtype can be used as important pathological factor predicting prognosis of breast cancer patients treated with anti-Her-2 therapy. Further analysis with a larger sample or a prospective study was needed to validate the conclusion.

## MATERIALS AND METHODS

### Ethical statement

The study was approved by the Institutional Review Board of Sun Yat-Sen University Cancer Center (Guangzhou, China).

### Patients and tissue samples

Between 1999 and 2010, 98 female patients who were diagnosed with primary breast cancer and received follow-up treatment at Sun-Yat-Sen University Cancer Center were included in the study. Tissue samples were collected post-operation but before chemotherapy and all the patients had provided written informed consent before collection. Clinicopathological information was shown in Table 1 including age at diagnosis, menopausal status, tumor size, nodal status, hormone receptor status, Her-2 status, TNM stages, chemotherapy regimens, etc. All patients received anti-Her-2 therapy.

### HE (hematoxylin and eosin) staining and evaluation of TILs

Full-face hematoxylin-and-eosin-stained sections were histopathologically evaluated for TILs. Intratumoral lymphocytes were defined as intraepithelial mononuclear cells within tumor cell nests or in direct contact with tumor cells and were reported as the percentage of the tumor epithelial nests that contained infiltrating lymphocytes. Stromal lymphocytes were defined as the percentage of tumor stroma area that contained a lymphocytic infiltrate without direct contact to tumor cells [6]. Two reviewers who were blind to clinical background performed histopathologic evaluation independently. For TILs, percent of lymphocytic infiltration was used to distinguish high or low level of TILs by above or below 10%.

### IHC staining and quantification of FOXP3+ Tregs, CD68+ Mφ and IL-17+ Th17

Formalin-fixed, paraffin-embedded tissue were sectioned into thick slices (5 μm) and then mounted on poly-L-lysine-coated adhesive slides. After drying at 60°C for 2 hours, tissue sections were dewaxed in xylene, then rehydrated through a graded ethanol series followed by 0.3% hydrogen peroxide in methanol for 10 min to inhibit endogenous peroxidase activity. After standard microwave heat epitope retrieval for 30 mins in citrate buffer solution, pH 6.0, samples were incubated with antibodies to FOXP3(1:100 dilution; Abcam, UK), CD68(1:800 dilution; R&D, USA), IL-17(1:150 dilution; ZSGB-Bio, China) for 12-14 hours at 4°C. Sections were subsequently incubated with an appropriate reagent from the EnVision™ Detection DAB kit (Dako) and counterstained with Harris hematoxylin.

Two reviewers who were blind to clinical background performed immunohistochemical evaluation independently. And FOXP3+ Tregs, CD68+ Mφ and IL-17+ Th17 in intratumoral and stromal site were firstly counted in 3 representative high-power fields respectively (HPF; x20 objective and x10 eyepiece), then the calculated average value of three counts>median was defined as patients with high numbers of FOXP3+ Tregs, CD68+ Mφ and IL-17+ Th17 in surgical specimens before chemotherpay. Definition of IHC positive cells of each marker in intratumoral and stromal sites was just as same as TILs in intratumoral and stromal sites. In some specimens, FOXP3 was also expressed in cancer cells and reported as the percentage of the tumor cells expressed it. The percent value>median was defined as patients with high FOXP3 expression in cancer cells.

### Statistical analyses

The association between count of FOXP3+ Tregs, CD68+ Mφ, IL-17+ Th17 and infiltration level of TILs with clinicopathological/biological features was examined using the chi-square test. Correlations between FOXP3+ Tregs, CD68+ Mφ, IL-17+ Th17 and infiltration level of TILs with OS were analyzed by Kaplan-Meier survival curves using the log-rank tests. Associations between count of FOXP3+ Tregs, CD68+ Mφ, IL-17+ Th17 and infiltration level of TILs and prognosis were assessed using the Cox proportional hazard model. All data were processed by SPSS 19.0 and P <0.05 was considered statistically significant.

## SUPPLEMENTARY TABLE




