# Supplementary material for: Tumor-infiltrating lymphocytes predict prognosis of breast cancer patients treated with anti-Her-2 therapy
Source: Oncotarget. 2016 Dec 23;8(3):5219–32. doi: 10.18632/oncotarget.14124 (PMC5354903; doi:10.18632/oncotarget.14124)
Supplement: Supplementary file 1 [file oncotarget-08-5219-s001.pdf]

Tumor-infiltrating lymphocytes predict prognosis of breast cancer patients treated with anti-Her-2 therapy

SUPPLEMENTARY TABLE

Supplementary Table S1: Quantification of FOXP3+ Tregs, CD68+ Mφ and IL-17+ Th17.

See Supplementary File 1
